# Downhill Running-Induced Muscle Damage in Trail Runners: An Exploratory Study Regarding Training Background and Running Gait

**DOI:** 10.3390/sports14010012

**Published:** 2026-01-04

**Authors:** Ignacio Martinez-Navarro, Juan Vicente-Mampel, Raul López-Grueso, María-Pilar Suarez-Alcazar, Cristina Vilar-Fabra, Eladio Collado-Boira, Carlos Hernando

**Affiliations:** 1 Physical Education and Sports Department, University of Valencia, 46010 Valencia, Spain; 2 Department of Physiotherapy, Medicine and Health Science School, Catholic University of Valencia, 46090 Torrent, Spain; juan.vicente@ucv.es; 3 Department of Education and Specific Didactics, University Jaume I, 12071 Castellon, Spain; grueso@uji.es (R.L.-G.); hernando@uji.es (C.H.); 4 Department of Nursery, University Jaume I, 12071 Castellon, Spain; malcazar@uji.es (M.-P.S.-A.); colladoe@uji.es (E.C.-B.); 5 Hematology and Hemotherapy Service, Castellon Provincial Hospital Consortium, 12002 Castellon, Spain; cristina.vilar@hospitalprovincial.es; 6 Sport Service, University Jaume I, 12071 Castellon, Spain

**Keywords:** lactate dehydrogenase, downhill running intervals, downhill running gait, vertical oscillation, step length

## Abstract

This study aimed to assess the effect of a downhill-running (DR) bout on muscle damage biomarkers. It also examined whether training background and gait kinematics may influence DR-induced muscle damage and strength loss. Thirty-six experienced trail runners (25 men, 11 women), participants of a 106 km ultra-trail, performed a 5 km DR bout at 15% decline and at an intensity equivalent to their first ventilatory threshold. Muscle damage biomarkers (creatine kinase, lactate dehydrogenase, and myoglobin) were analyzed before and 30 min after the DR protocol, and also before and after the UT race. Isometric strength was assessed before and after DR, and gait parameters were recorded during DR. All muscle damage biomarkers increased following DR (*d* = 0.19 to 1.85). Lactate dehydrogenase concentrations after the race and DR were associated (*r* = 0.64). Athletes who habitually performed downhill repetitions showed reduced creatine kinase (182 ± 73 U/L vs. 290 ± 192 U/L; *p* < 0.05; d = 0.64) and greater squat strength retention (4 ± 10% vs. −9.1 ± 16.8%; *p* <0.05; *d* = 0.87). Ankle plantar flexion and squat strength retention were inversely correlated with vertical oscillation (*r* = −0.44) and step length (*r* = −0.37), respectively. In summary, lactate dehydrogenase response to a short DR bout could indicate an athlete’s readiness to handle ultra-trail-induced muscle damage, although further research is needed to confirm it. In addition, despite the exploratory nature of the study, regularly performing downhill intervals and adopting a more terrestrial gait pattern appear to soften strength loss and muscle damage response to DR.

## 1. Introduction

The rate of speed decrease during an ultra-trail (UT) race is usually greater on descents and flat sections than on ascents [1]. Minimizing speed loss on downhill sections toward the end of UT races appears to be a trait of top performers [2]. It has been suggested that faster finishers can maintain greater relative performance in downhill sections not only because of their superior aerobic capacity, but also, most importantly, because of less exercise-induced muscle damage (EIMD) and fatigue [2]. In view of this perspective, slower runners would self-regulate to a lesser intensity in downhill sections to prevent more severe musculoskeletal damage and reduce pain perception [2,3].

After the Ultra Trail du Mont-Blanc or Western States Endurance Run, mean post-race creatine kinase (CK) concentrations can exceed 15,000 U/L [4,5]. Much of this muscle damage occurs during downhill sections, as the eccentric phase of the stretch-shortening cycle is intensified [6]. Muscle damage is thus proposed as a main performance-limiting factor in mountain running [7,8,9]. Even in flat marathons, greater resistance to EIMD may help maintain speed in the second half of the race, as runners who slow down the most tend to show the greatest post-race muscle damage, while those with more even pacing have reduced muscle damage markers [10]. Indeed, it has recently been recommended to assess muscle damage from downhill running (DR) to gauge athletes’ tolerance to UT-specific muscle damage and explore training strategies to mitigate it [11].

Prior exposure to DR has been shown to reduce markers of muscle damage and strength loss, creating a repeated bout effect (RBE) in untrained individuals and recreational runners [12,13]. Four weeks (10 sessions) of DR training in previously untrained individuals also promoted neuromuscular adaptations typical of high-intensity eccentric resistance training [14]. However, it is unclear whether regularly performing DR intervals in experienced trail runners (TR) provides extra protection against muscle damage and strength impairment. In experienced TR, high-intensity DR causes strength losses in knee extensors and plantar flexors similar to those after a UT race [15], and much greater than those after uphill running at similar oxygen uptake [16]. Therefore, trail runners who regularly perform DR repetitions may experience less muscle damage and strength loss after DR. Strength training, especially isometric exercises at long muscle lengths, has shown similar protective effects in untrained individuals [17,18]. No studies, however, have examined this effect in experienced trail runners.

The aim of this study was four-fold: (i) to assess the immediate effect of a DR bout on muscle damage biomarkers in experienced TR; (ii) to explore whether muscle damage after a 5 km DR bout correlates with damage after a UT race; (iii) to explore whether DR-induced muscle damage and strength loss differ among athletes who typically perform strength training and DR intervals; (iv) to explore possible links between DR-gait and DR-induced muscle damage and strength loss. Our hypotheses were: (1) muscle damage following a 5 km DR bout would correlate with that after a UT race; (2) athletes who regularly perform strength training and downhill repetitions would suffer less post-DR strength loss and muscle damage; (3) DR-induced muscle damage and strength loss would be associated with smaller step length and lower vertical oscillation.

## 2. Materials and Methods

### 2.1. Participants

Considering the effect size found in a previous work (large, >0.8) who assessed the change in plasma myoglobin (Mb) immediately after a DR protocol similar to ours [19], a sample size of 19 participants was deemed appropriate to find significant within-group differences in the present study (1-tailed α < 0.05, 1-β > 0.95) (Gpower, version 3.1.9.7, Universität Düsseldorf, Düsseldorf, Germany). Thirty-six experienced TR (25 men and 11 women) finally joined the study. The inclusion criteria were: participation in the 2025 Penyagolosa Trails CSP race and completion of at least two races longer than 65 km. The exclusion criteria were: having any cardiac or renal disease and taking any medication on a regular basis. The research took place in the months leading up to and during the 2025 Penyagolosa Trails CSP race. The racetrack was 106.1 km long, starting at 65 m and ending at 1280 m above sea level, with total positive and negative elevations of 5584 m and 4369 m, respectively. All participants were fully informed about the procedure and provided written consent. Participation was voluntary, with the option to withdraw at any time. A questionnaire was used to collect demographic, training, and competition information. The investigation was conducted in accordance with the Declaration of Helsinki and received approval from the Research Ethics Committee of the University Jaume I of Castellon (reference number CEISH/103/2024). The study was registered at ClinicalTrails.gov with code NCT06969898 (www.clinicaltrials.gov, accessed on 5 May 2025).

### 2.2. Experimental Overview

This study was part of a larger research project. In brief, participants visited the laboratory twice, two weeks apart. Body composition was assessed at the start of each visit. On the first visit, 6–8 weeks before the race, participants completed a cardiopulmonary exercise test (CPET) and familiarized themselves with the downhill protocol scheduled for the second visit. The second visit, 4–6 weeks prior to the race, began with a blood draw and muscle morphology assessment, followed by isometric strength tests and an uphill walking economy test. Participants then performed the DR protocol. Afterward, they repeated the isometric strength and uphill walking economy tests. Muscle morphology was reassessed, and a second blood sample was collected after the DR. This paper focuses on muscle damage biomarkers, training background, and DR-gait kinematics data.

Participants attended both visits after fasting for more than six hours and maintained their regular mixed macronutrient diet the day prior to testing. Body Mass Index (BMI), fat mass percentage (%FM), and lean body mass percentage (%LBM) were assessed using a bioelectrical impedance weight scale (Tanita BC-780MA, Tanita Corp., Tokyo, Japan). Measurements were taken while participants wore minimal clothing (running shorts and a t-shirt), in accordance with the manufacturer’s guidelines. Skin and electrodes were thoroughly cleaned and dried before assessment. On the second visit, prior to the DR protocol, participants consumed an energy bar or gel containing 60 g of carbohydrates [20]. Resistance training, DR, and vigorous running were prohibited for 48 h before testing, and any training was not allowed within 24 h. Upon arrival at the laboratory, all pre-trial standardization procedures were confirmed verbally with each participant.

### 2.3. Cardiopulmonary Uphill Incremental Test

CPETs were performed on a treadmill (SK7990, BH Fitness, Vitoria, Spain) with a 20% constant slope. After a 3 min warm-up at 2.5 km·h^−1^, corresponding to a vertical velocity of 500 m·h^−1^, speed was increased 0.5 km·h^−1^ every minute (equivalent to a vertical velocity of 100 m·h^−1^) until volitional exhaustion [21]. We opted for a lower gradient than suggested in the original protocol (25%), that it was tested in highly trained males, because our sample consisted of males and females with greater heterogeneity in fitness levels. Accordingly, we aimed to avoid premature muscle fatigue that could, in turn, hinder the attainment of maximal oxygen uptake (VO_2_max). Participants were free to walk or run as they preferred. Expired gases and heart rate were collected continuously using indirect calorimetry (Quark CPET, COSMED^®^, Rome, Italy) and a chest-strap heart rate monitor (H10, Polar Electro Oy, Kempele, Finland). The gas analysis system was calibrated (including replacing the sample line and turbine) before each test to improve stability and sensitivity of the instrumentation [22]. VO_2_max values were accepted when a plateau (an increase of <2 mL/kg/min) or a decline in VO_2_ was reached despite increasing workloads. If these criteria were not met, a VO_2_peak value was taken, defined as the greatest VO_2_ measured over a 30 s period. First and second ventilatory thresholds (VT_1_ and VT_2_) were determined using the guidelines of Skinner and McLellan [23] by two independent researchers. Peak vertical velocity (V_vert_peak) and vertical velocities corresponding to VT_1_ and VT2 (V_vert_VT_1_, V_vert_VT_2_) were calculated as the speed of the last complete stage added to the multiplication of the speed increment by the completed fraction of the incomplete stage [24].

### 2.4. Downhill Running Protocol

The DR protocol consisted of 5 km at 15% constant decline, with the intention of mimicking descents typical of major UT races [11]. Negative gradient was achieved by elevating the rear part of the treadmill with a custom-made platform and verifying the inclination using a goniometer. Two researchers were on each side of the treadmill throughout the test as a safety precaution to minimize the risk of falling. During the first visit, following the end of the CPET, the speed for the DR protocol was individually set to coincide the HR with the HR at uphill VT_1_. Briefly, participants walked at 6 km·h^−1^ for 1 min, ran at 10 km·h^−1^ for 3 min, and afterwards speed was set at 300% of their uphill V_vert_VT_1_. This reference was based on pilot work and previous studies indicating that VT_1_ on a −15% gradient was ≈2.8 times greater than at a 15% gradient in trained TR [25]. After a 1 min stabilization period for HR, speed was manually adjusted until the HR coincided (±5%) with the one at uphill VT_1_. This procedure also served as a familiarization for the second visit test. ‘Downhill-adjusted’ VT_1_ speed was 13.3 ± 1.6 km·h^−1^ (325 ± 9% of uphill VT_1_ speed). Step length (SL), step frequency (SF), ground contact time (GCT), and vertical oscillation (VO) were measured using the Stryd powermeter (Stryd Inc., Boulder, CO, USA) [26,27,28].

### 2.5. Isometric Ankle Plantar Flexion and Half-Squat Strength Tests

Participants were familiarized with procedures concerning strength assessment during an informative session prior to the investigation. Isometric maximal voluntary contraction (MVC) was measured with a force sensor (Chronojump, Barcelona, Spain) [29] held onto a bar using a custom-adapted Smith machine. Bar height for each participant and test was individually set and firmly anchored to the ground using chain and pins to impede any movement. For the ankle plantar flexion (PF) assessment, participants stood upright with their hips and knees fully extended and their ankles in a 0-degree position of plantar flexion. For the half-squat (SQ) assessment, they adopted a knee angle of 140 degrees in the ready position [30]. Each test was performed twice, and the best performance was retained for statistical analysis. A 1 min rest was provided between attempts. For each contraction, participants were instructed to exert maximal upward force against the bar, mimicking the pattern of a half-squat and a calf extension, respectively. Each contraction was initiated following a verbal prompt from the researcher, and consistent verbal encouragement was provided throughout the process. The maximum force produced was modeled using the inverse monoexponential function that better fitted the raw data. This fitting was made by adjusting the maximum force to the speed at which the maximum force was reached. This method was used to more accurately estimate MVC, accounting for variations in the rate of force development and potential signal noise, as per [31].

### 2.6. Blood Sampling and Analysis

Blood samples were collected from an antecubital vein by venipuncture. Collection was performed by experienced nurses using BD Vacutainer PST II tubes on four occasions: before and 30 min after the DR protocol -as previously suggested [11]-, before and 30 min after the race. Samples were centrifuged at 3500 rpm for 10 min and maintained at 4 °C during transport to the Castellon Provincial Hospital Consortium, where they were processed using a Beckman Coulter DXC 700 AU analyzer (Beckman Coulter, Inc., Brea, CA, USA) [32]. The following blood variables were considered for analysis: lactate dehydrogenase (LDH), CK, and Myoglobin (Mb). Post-race values were adjusted using the Dill and Costill method [33], which employed hematocrit and hemoglobin to determine the magnitude of plasma volume changes in each participant after the race [33,34].

### 2.7. Statistical Analysis

Statistical analyses were performed using the Statistical Package for the Social Sciences software (IBM SPSS Statistics for Windows, version 30; IBM Corp., Armonk, NY, USA). Normality was checked using the Shapiro–Wilk test, and all variables met normality assumptions. Paired-samples *t*-tests were used to assess the effect of DR on muscle damage biomarkers (CK, LDH, and Mb). The pre-to-post DR change in PF, SQ, LDH, CK, and Mb was compared between participants who usually perform strength training (a minimum of 2 sessions a week) and downhill repetitions (a minimum of 1 session a week), and those who do not during the 3 months prior to the race, using unpaired-samples *t*-tests. Homogeneity of variance was verified by Levene’s test. CK values distribution as a function of downhill repetitions engagement did not meet the equality of variances assumption. Accordingly, a one-way ANOVA with the Welch statistic was used in that case. Lastly, Pearson product-moment correlations were computed to explore: (1) whether DR-induced muscle damage was correlated with post-race muscle damage in the finishers sample set; (2) whether muscle damage and strength loss (ΔPF and ΔSQ) provoked by DR were associated with running gait. The meaningfulness of the outcomes was estimated through Cohen’s d effect size and 95% confidence intervals (CIs). A Cohen’s d < 0.5 was considered small; between 0.5 and 0.8, moderate; and greater than 0.8, large. Likewise, correlations > 0.5 were considered strong, 0.3–0.5, moderate, and <0.3, small. The significance level was set at *p* < 0.05, and data are presented as means and standard deviations (±SD).

## 3. Results

Participants’ characteristics, including demographic information, training and competition history, and data from the CPET, are presented in Table 1. All muscle damage biomarkers significantly increased from pre- to post-DR (Table 2). The change in CK and LDH was small in magnitude, while the rise in Mb was large.

No significant differences were observed in post-DR CK (268 ± 191 U/L vs. 256 ± 119 U/L; *p* = 0.877; d = 0.07; 95% CI, −0.76 to 0.90), LDH (430 ± 82 vs. 427 ± 58 U/L; *p* = 0.915; d = 0.05; 95% CI, −0.78 to 0.88) and Mb (98 ± 51 vs. 75 ± 18; *p* = 0.239; d = 0.52; 95% CI, −0.32 to 1.36) between those participants who usually performed strength training and those who not. Similarly, no significant differences appeared in SQ (−4.4 ± 17.3% vs. −13.3 ± 10.3%; *p* = 0.202; d = 0.57; 95% CI, −0.27 to 1.41) and PF (−12.4 ± 15.6% vs. −13.4 ± 16%; *p* = 0.883; d = 0.06; 95% CI, −0.76 to 0.89) strength loss following DR between those participants who usually performed strength training and those who not. Neither significant differences were noted in LDH (427 ± 58 U/L vs. 430 ± 82 U/L; *p* = 0.939; d = −0.03; 95% CI, −0.82 to 0.76), Mb (74 ± 40 U/L vs. 99 ± 48; *p* = 0.191; d = −0.55; 95% CI, −1.36 to 0.25) and PF strength loss (−12 ± 14.7% vs. −12.9 ± 15.9%; *p* = 0.895; d = 0.06; 95% CI, −0.74 to 0.85) between those participants who habitually performed downhill repetitions and those who not. However, those athletes who habitually performed downhill repetitions showed lesser CK (182 ± 73 U/L vs. 290 ± 192 U/L; *p* < 0.05; d = 0.64; 95% CI, −1.44 to 0.17) and greater SQ strength retention (4 ± 10% vs. −9.1 ± 16.8%; *p* < 0.05; d = 0.87; 95% CI, 0.05 to 1.69) after the DR protocol.

**Figure 1:**
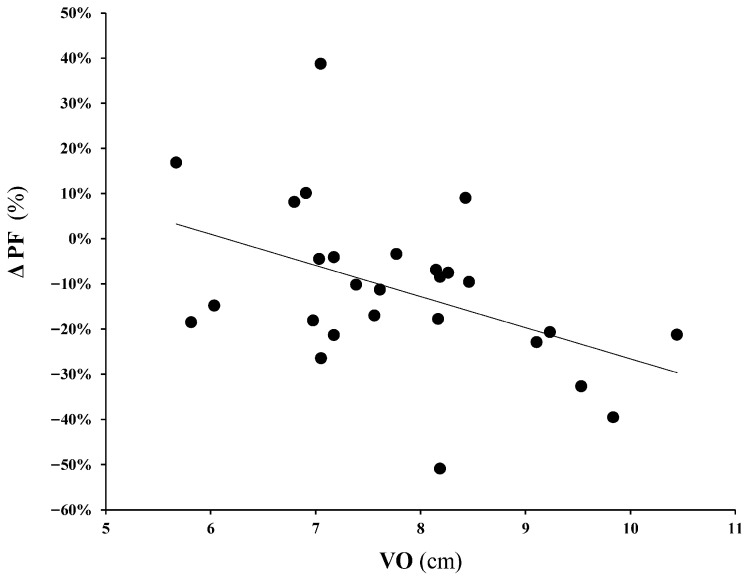
Relationship between pre-to-post downhill running change in PF and VO during the downhill running protocol.

**Figure 2:**
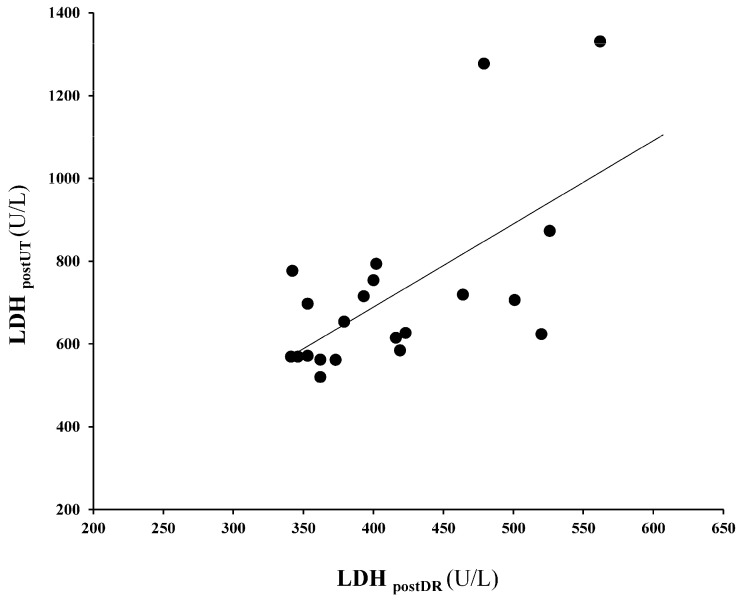
Relationship between LDH concentrations post-DR and post-UT race.

No relationships were found between DR gait variables and muscle damage biomarkers. However, ΔPF was moderately and significantly inversely correlated with VO (r = 0.44; *p* < 0.01) (Figure 1) and ΔSQ was moderately and significantly inversely correlated with SL (r = 0.37; *p* < 0.05). From the initial sample (36 athletes), 1 participant did not start the race due to injury, and 27 athletes (21 men and 8 women) successfully completed the race. In that sample set, correlational analysis revealed that LDH concentrations following the race and the DR protocol were strongly associated (r = 0.64; *p* < 0.01) (Figure 2). Notwithstanding, two participants exhibited exceptionally large LDH values post-race, and the correlation fell to moderate when their data were not considered (r = 0.429; *p* = 0.032). Conversely, in the case of CK and Mb, post-DR and post-UT levels were not correlated. Pre-to-post race values of CK, LDH, and Mb were 146 ± 68 vs. 3930 ± 3522 U/L, 346 ± 50 vs. 725 ± 201 U/L, and 25 ± 13 vs. 1107 ± 829 ng/mL, respectively.

## 4. Discussion

Muscle damage and its associated fatigue have been argued to be a main impediment to performance in UT races [7,8,9,11]. Much of this muscle damage occurs during downhill sections [6], and the speed decay is more pronounced in those sections of UT races [1,2]. No studies, however, had previously assessed DR-induced muscle damage in a sample of experienced TR. Our purposes were to explore whether DR-induced muscle damage was correlated with UT-induced muscle damage, and whether DR-induced muscle damage differed as a function of training background and DR-gait. The results of the study showed that even among experienced TR, a 5 km DR bout (750 m of cumulative elevation loss) performed at light cardiovascular intensity (equivalent to VT_1_, 13.3 ± 1.6 km·h^−1^), provokes a significant rise in muscle damage biomarkers. CK and LDH showed an immediate response of small magnitude (6 to 17% increase, d = 0.19 and 0.30, respectively), but Mb evidenced a large increase (248%; d = 1.85). Regarding the correlational analyses, on the one hand, athletes who typically performed DR intervals in their training regimen (at least once a week) exhibited reduced CK and greater SQ resilience after the DR protocol. On the other hand, a more terrestrial DR pattern (i.e., lower VO and SL) was moderately associated with a greater strength retention, thus confirming our second hypothesis. Lastly, in accordance with our third hypothesis, LDH after the DR bout and the UT race were correlated, although the magnitude of the association fell from strong to moderate when post-race extreme values were not considered.

Training status and modality are known to reduce EIMD [6], and our sample consisted of experienced TR (15 ± 8 years of running training) who cumulate a weekly average of 2514 ± 1132 m of positive/negative elevation. Consequently, a more attenuated response of muscle damage biomarkers was expected as compared with previous studies conducted in road runners or untrained individuals [13,19,35]. Meanwhile, blood levels of CK and LDH rise at a slower rate than Mb levels, who has a faster response to EIMD (i.e., rise and reach their peak in the blood earlier) [36]. Indeed, Mb has been shown to be more sensitive than CK and LDH as a marker of EIMD in the immediate recovery [36,37]. Consistent with this previous literature, our results showed that Mb increased following the DR bout to a larger magnitude than CK and LDH. On the other hand, although further studies with larger samples are needed to confirm it, our results support the suggested appropriateness of measuring DR-induced muscle damage following a standardized protocol (i.e., 5 km at 15% decline) to calibrate TR readiness to sustain UT-induced muscle damage [11]. The absence of a similar relationship in CK and Mb was probably due to their greater interindividual variability.

Despite the exploratory nature of the study, our results suggest that incorporating DR intervals into the training regimes of TR is suitable, as reduced CK concentration and greater strength retention were observed in participants with this training background. Accordingly, DR intervals appear to be a promising training tool for reducing speed decay at the latter stages of competition, thereby enabling a more even race pace [10]. Research regarding the effects of DR training is scarce. Eight weeks (16 sessions) of downhill interval training (10% decline) in physically active individuals improved squat jump performance and knee-extensor torque [38]; meanwhile, four weeks (10 sessions) of DR training (5 to 15% decline) in previously untrained individuals enhanced knee-extensor torque, morphology, and architecture [14]. Nevertheless, to our knowledge, no studies have been conducted with trained runners. Thereby, further intervention studies are needed to verify whether the application of such training sessions improves the resilience of TR to DR-induced muscle damage and loss of strength.

DR gait has been demonstrated to be an important factor determining the magnitude of induced muscle damage and strength impairment [6,39,40,41,42]. Rowlands et al. [40] reported lesser strength loss and perceived muscle soreness following an intermittent 45 min DR protocol (15% decline) when a reduced step length (i.e., by increasing step frequency) was adopted. Similarly, Baggaley et al. [39] demonstrated that reducing step length during DR (5 and 10% decline) diminished knee joint energy absorption and impact attenuation. In line with those previous studies, our results showed that a more terrestrial gait pattern during DR (i.e., lower VO and SL) was associated with greater strength retention. Those changes in running biomechanics during UT races have been suggested as a compensatory response to attenuate pain perception due to muscle damage [43,44,45]. Therefore, it is possible that previous pain experiences associated with DR led some participants to adopt a more terrestrial gait pattern during the DR protocol in an anticipatory manner [3,46].

We acknowledge the exploratory nature of the study. This design prevented us from clearly distinguishing the possible protective effects of DR training from those of strength training. Indeed, 7 participants were engaged in both downhill repetitions and strength training, and this could represent a potential source of bias in the study.

## 5. Conclusions

LDH concentration after a short DR bout (5 km at 15% decline) was strongly correlated with LDH following a UT race, so it might constitute a feasible indicator of an athlete’s preparedness to face UT-induced muscle damage. Notwithstanding, further studies with larger samples are needed to confirm this suggestion. Even in experienced trail runners, accustomed to graded running, a short DR bout performed at light cardiovascular intensity provoked a significant immediate rise in muscle damage biomarkers. Regularly undertaking DR intervals and adopting a more terrestrial gait pattern (i.e., lower VO and SL) appears to soften strength loss and muscle damage response to DR.

Thereby, current results suggest that coaches consider incorporating DR intervals in the training programs of TR to increase their tolerance to eccentric contractions. At the same time, adopting a more terrestrial DR gait pattern during prolonged training sessions and competitions is recommended to attenuate muscle damage and loss of strength, and to facilitate a better recovery. On the other hand, both coaches and clinicians are encouraged to assess LDH response to DR as a marker of TR resilience against eccentric-induced muscle damage. Further research is required to corroborate whether the application of the above recommendations improves TR performance in UT races.

## Figures and Tables

**Table 1:** Sample main characteristics (mean ± SD).

	All Sample (*n* = 36)	Males (*n* = 25)	Females (*n* = 11)
Age (years)	45 ± 8	44 ± 6	49 ± 9
Number of years running	15 ± 8	14 ± 7	15 ± 8
Number of races > 100 km	3 ± 4	4 ± 5	2 ± 2
Weekly training days	5 ± 1	5 ± 1	5 ± 1
Weekly running volume (km)	71 ± 22	70 ± 22	74 ± 23
Weekly cumulative positive/negative elevation (m)	2514 ± 1132	2651 ± 1233	2172 ± 780
Weekly training hours	13 ± 6	13 ± 7	12 ± 4
Strength training (%)	75.7%	76%	75%
Downhill repetitions (%)	21.6%	24%	16.7%
BMI (kg/m^2^)	23 ± 2.4	23.7 ± 2.2	21.5 ± 2.2
Height (cm)	171 ± 8	177 ± 5	161 ± 4
FM (%)	18.5 ± 6.4	17.1 ± 6	21.8 ± 6.6
LBM (%)	78.3 ± 6	80 ± 5.5	74.3 ± 5.5
V_vert_VT_1_ (m/h)	820.8 ± 97.6	853.2 ± 87	746.9 ± 80.9
%VT_1_ (% VO_2_peak)	65.2 ± 5.7	63.8 ± 5.5	68.5 ± 5.1
V_vert_VT_2_ (m/h)	1086.9 ± 140.4	1135.7 ± 134.8	976 ± 76.6
%VT_2_ (% VO_2_peak)	82.8 ± 7.1	82.2 ± 7	84.2 ± 7.7
VO_2_peak (ml O_2_/kg/min)	57 ± 8	59 ± 7.9	52.4 ± 6.6
V_vert_peak (m/h)	1442.9 ± 182.2	1514.6 ± 159.7	1279.9 ± 113.7

Abbreviations: Strength training (%), percentage of participants who perform at least two weekly lower-limb strength training sessions; Downhill repetitions (%), percentage of participants who perform downhill running intervals at least one weekly; BMI, Body mass index; FM, fat mass; LBM, lean body mass; V_vert_VT_1_, speed at the first ventilatory threshold; %VT_1_, percentage of VO_2_peak at the first ventilatory threshold; V_vert_VT_2_, speed at the second ventilatory threshold; %VT_2_, percentage of VO_2_peak at the second ventilatory threshold; VO_2_peak, peak oxygen up-take; V_vert_peak, peak speed reached at the CPET.

**Table 2:** Muscle damage biomarkers before (PRE) and after (POST) the downhill running protocol (mean ± SD).

	Pre	Post	Change from Pre, %	Cohen d; 95% CI
CK (U/L)	229 ± 168	261 ± 177 *	17.4 ± 10.2%	0.19; −0.27 to 0.65
LDH (U/L)	405 ± 78	428 ± 75 *	6.4 ± 9.9%	0.3; −0.16 to 0.77
Mb (ng/mL)	31 ± 16	94 ± 47 *	247.8 ± 229.3%	1.85; 1.3 to 2.4

Abbreviations: CK, creatine kinase; LDH, lactate dehydrogenase; Mb, myoglobin. * Significantly different from pre (*p* < 0.01).

## Data Availability

The data presented in this study are available on request from the corresponding author due to ethical reasons.

## Abbreviation

The following abbreviations are used in this manuscript

%FM	Percentage of fat mass
%LBM	Percentage of lean body mass
%VT_1_	Percentage of VO_2_peak at the first ventilatory threshold
%VT_2_	Percentage of VO_2_peak at the second ventilatory threshold
BMI	Body Mass Index
CPET	Cardiopulmonary exercise test
CK	Creatine kinase
DR	Downhill running
EIMD	Exercise-induced muscle damage
GCT	Ground contact time
FR	Frequency of respiration
HR	Heart rate
LDH	Lactate dehydrogenase
Mb	Myoglobin
PF	Isometric ankle plantar flexion strength
SF	Step frequency
SL	Step length
RBE	Repeated bout effect
SQ	Isometric half-squat strength
TR	Trail runners
UT	Ultra-trail
UW	Uphill walking
VE	Minute ventilation
VO	Vertical oscillation
VO_2_max	Maximal oxygen uptake
VO_2_peak	Peak oxygen uptake
VT	Tidal volume
VT_1_	First ventilatory threshold
VT_2_	Second ventilatory threshold
V_vert_peak	Peak vertical speed reached at the cardiopulmonary exercise test
V_vert_VT_1_	Vertical speed at the first ventilatory threshold
V_vert_VT_2_	Vertical speed at the second ventilatory threshold
